# Human Complement Evasion Is Widespread Among Lyme Borreliosis Spirochete Species

**DOI:** 10.3390/pathogens15070717

**Published:** 2026-07-07

**Authors:** Maryna Golovchenko, Lucie Krätzerová, Heather MacTavish, Vett Lloyd, Natalie Rudenko

**Affiliations:** 1 Institute of Parasitology, Biology Centre CAS, 370 05 Ceske Budejovice, Czech Republic; marina@paru.cas.cz; 2 Biological Defence Department Techonin, Military Health Institute, 561 66 Techonin, Czech Republic; lu.ticha@seznam.cz; 3 Department of Biology, Mount Allison University, Sackville, NB E4L 1G7, Canada; hrmactavish@mta.ca

**Keywords:** *Borrelia*, Lyme borreliosis, Lyme disease, human complement, infectivity, immune response, complement-mediated killing, host sex and age

## Abstract

Disseminated human Lyme borreliosis is primarily associated with invasive spirochetes from the *Borrelia burgdorferi* sensu lato complex. Host–*Borrelia* interactions have been studied in a diverse range of vertebrate reservoirs. But despite the key role of the complement system in innate immunity, comparative studies evaluating the susceptibility of individual *Borrelia* species to human complement-mediated killing are limited. Using serum sensitivity assays, we analyzed complement-mediated killing of 10 *B. burgdorferi* s.l. genospecies in healthy individuals of different ages and sexes. The tested genospecies showed markedly different sensitivities to human complement. Statistical clustering divided the genospecies into three distinct groups based on whether their sensitivity to human complement was high, medium, or low. Complement resistance did not correlate with the recognized pathogenicity of the genospecies; species with unclear pathogenic potential exhibited resistance levels comparable to the major species causing human Lyme borreliosis, *B. burgdorferi* sensu stricto, *B. afzelii* and *B. garinii*. Females generally showed reduced complement-mediated killing compared to males. In addition, complement activity tended to decline with age, identifying host age and biological sex as important factors influencing the human innate immune response against *Borrelia*. Our findings suggest that the ability to evade human complement may be more widespread among *Borrelia* species than previously assumed. These results support an emerging model in which complement-mediated selection may shape host associations and transmission success without serving as an absolute predictor of *Borrelia* virulence.

## 1. Introduction

The *Borrelia burgdorferi* sensu lato complex currently consists of 23 spirochete species worldwide, along with two recently proposed South American species [1,2]. Four of these species, *B. afzelii*, *B. burgdorferi* sensu stricto (s.s.), *B. bavariensis* and *B. garinii*, are the major confirmed causes of Lyme borreliosis (LB), a multisystem disorder with a diverse spectrum of clinical manifestations. However, pathogenicity of other *Borrelia* species to humans, particularly *B. bissettiae*, *B. lusitaniae*, *B. mayonii* and *B. spielmanii*, has also been described [1,3,4]. Members of the *B. burgdorferi* s.l. complex are often divided based on human clinical infections; one group includes species with documented isolation from humans, while the other group consists of species that have not yet been isolated from, or detected in, humans. However, the distinction between pathogenic and non-pathogenic *Borrelia* species may change as less well-studied species are further characterized. The group of *Borrelia* isolated or detected in humans includes 12 species: *B. afzelii*, *B. americana*, *B. andersonii*, *B. bavariensis*, *B. bissettii*, *B. burgdorferi* s.s., *B. garinii*, *B. kurtenbachii*, *B. lusitaniae*, *B. mayonii*, *B. spielmanii* and *B. valaisiana* [1,4,5,6]. The remaining 11 species that have not been detected in humans are: *B. californiensis*, *B. carolinensis*, *B. chilensis*, *B. finlandensis*, *B. japonica*, *B. lanei*, *B. maritima*, *B. tanukii*, *B. turdi*, *B. sinica*, and *B. yangtzensis* [1,4,5,6]. Among the species that have been detected in humans, the frequency of recovery varies greatly. While *B. afzelii, B. bavariensis, B. garinii* or *B. burgdorferi* s.s. are commonly detected in diagnosed cases of LB in endemic regions in the Northern Hemisphere [1], a limited number of patients have tested positive for *B. bissettii*, *B. kurtenbachii*, *B. mayonii*, *B. spielmanii*, or *B. valaisiana*, and human cases involving *B. americana, B. lusitaniae*, or *B. andersonii* are only rarely documented [3,7]. What causes the unequal recovery of different *Borrelia* species from humans? Humans are accidental hosts for tick vectors, so exposure depends on a complex interplay of ecological and human behavioral factors. However, when a human is exposed to a specific spirochete species, what determines whether *Borrelia* can disseminate and cause disease? Is the predominant detection of only a few *Borrelia* species due to biological constraints on the pathogenicity of each species, or is it the result of a historical research focus on *B. burgdorferi* s.s., *B. afzelii*, and *B. garinii* to the exclusion of other species?

Spirochetes first encounter the host immune system upon entering the skin and subsequently in the interstitial fluid, bloodstream or lymphatic system as they travel to other tissues. When spirochetes contact complement in these tissues, the innate immune response is activated [8,9]. As a critical component of the innate immune system, the complement cascade consists of a series of serum proteins that, upon pathogen recognition, initiate a defensive signaling cascade. This response drives opsonization, inflammatory signaling, and membrane attack complex-mediated lysis while simultaneously recruiting and enhancing the adaptive immune response [10,11]. At the beginning of infection, before pathogen-specific antibodies are generated, the invading spirochetes are attacked by the non-specific alternative complement pathway [12,13]. *Borrelia* produces several complement regulator-acquiring surface proteins (CRASPs), BbCRASP-1 to BbCRASP-5, that bind regulatory proteins of the alternative complement pathway, including factor H (FH) and factor H-like protein 1 (FHL-1), a truncated alternatively spliced variant of FH [13]. Major *Borrelia* CRASPs belong to different gene families and have different binding profiles. CRASP-1 (CspA; PFam54 family) and CRASP-2 (CspZ, unique family) bind both FH and FHL-1 [12,13,14,15]. The other three proteins, CRASP-3 (ErpP), CRASP-4 (ErpC), and CRASP-5 (ErpA), belong to the OspE/F-related (Erp) protein family and recognize different regions on the C-terminus of factor H that are missing from FHL-1 so they bind FH only and do not interact with FHL-1 [12,13,14,15]. The differential binding profile of *Borrelia* CRASPs suggests a sophisticated mechanism by which the spirochete utilizes multiple surface proteins to neutralize complement activation and avoid recognition and eradication by the host complement system. Just like FH, FHL-1 is an essential regulatory component of the vertebrate host’s innate immune system [16]. In host defense against *Borrelia*, the alternative pathway plays a particularly important role [17]. It has been found that *Borrelia*’s host range is determined in both wild and laboratory animals, in part, by its sensitivity to the complement of a particular vertebrate host species [8,9,18,19,20,21,22,23,24], as the ability to bind FH and FHL-1 depends on the *Borrelia* species [8,16]. As a result, this divides spirochete species into serum-sensitive and serum-resistant strains. Here, we investigate whether differences in serum sensitivity are associated with variation in human pathogenic potential among diverse *Borrelia* species.

## 2. Materials and Methods

### 2.1. Cultivation of Borrelia Species

Ten *Borrelia* species, *B. afzelii* CB43 (Czech isolate, a gift from J. Kopecky), *B. burgdorferi* s.s. B31, *B. andersonii* 21038, *B. bissettiae* DN127, *B. kurtenbachii* 25015 (a gift from the late James H. Oliver Jr. spirochete collection), *B. carolinensis* SCGT18, *B. americana* SCW30F, *B. garinii* 20047, *B. bavariensis* PBi and *B. valaisiana* VS116 were selected and cultured in modified Barbour–Stoenner–Kelly medium (BSK-H) under sterile conditions as described earlier [25,26]. No antibiotics were used in spirochete re-cultivation. Newly seeded cultures were incubated at +34 °C with regular monitoring of the growth by dark-field microscopy. Identity of the *Borrelia* species in the culture was confirmed by PCR (*rrf-rrl* intergenic spacer, 16S rRNA, *flaB*, *ospA*, and p66 genes) on genomic DNA isolated from a culture aliquot using the DNeasy Blood and Tissue kit (Qiagen, Berlin, Germany) as described [26]. Cultures were grown until density reached 10^7^ cells/mL (from 5 to 8 days) and were then used in the serum sensitivity test. All species included in this study represented low-passage (3–5 passages) strains.

### 2.2. Human Serum Samples

To obtain the human samples, the office of a general practitioner physician, where patients’ blood was collected for routine healthcare on a daily basis, was contacted. During the blood collection all patients were informed about the opportunity to provide residual blood samples (2 mL) for research, and verbal consent was obtained; 148 blood samples were collected in this way; two donors declined participation. The blood samples provided to the research team were anonymous, with only age and biological sex provided for each sample, and no record connecting blood samples to patient identity was made. No information on health status of donors, medication use, their infection history, pregnancy, immunosuppressive treatment, or complement deficiencies was obtained. Seropositive samples with anti-*Borrelia* antibodies (8 male and 2 female from people 41–60 years old) were excluded following screening by the rapid immunochromatographic test (Lymetest (Lyme IgG/IgM rapid test for whole blood/serum/plasma), Biorepair, Bielefeld, Germany). Processing of all collected samples was done in the laboratory by a single person immediately upon collection of donated blood. The blood samples were centrifuged at 800 *g* at room temperature for 15 min to separate blood cells and serum and then filtered through a 0.22 μm sterile PES syringe filter (Fisher Scientific, Waltham, MA, USA). Samples were divided into 6 age categories of donors: category 1 (21–30 years), 2 (31–40 years), 3 (41–50 years), 4 (51–60 years), 5 (61–70 years) and 6 (over 71 years). Each category was further divided into samples from i) biological females and ii) biological males. All samples were stored at −80 °C until collection of samples was completed (1 month). Ethical consent to conduct this study was provided by the Ethics Committee of the Biological Center in České Budějovice (5/2024) and the Mount Allison University Research Ethics Board (104186). The goal of this study was to evaluate and compare the relative susceptibility of various species within the *B. burgdorferi* s.l. complex to complement-mediated killing when exposed to serum derived from healthy individuals of varying ages and biological sexes.

### 2.3. Flow Cytometric Assessment of Borrelia Membrane Disruption

Complement sensitivity testing was conducted according to the protocol established by Kurtenbach et al. [19] with slight modifications and the use of flow cytometry rather than manual darkfield microscopy, necessary to support the sample size of this study. Earlier studies confirmed that flow cytometry is a rapid method for determining the presence of borreliacidal activities, including complement-mediated killing [27,28], by distinguishing membrane-intact (live) from membrane-compromised (dead) bacteria. This methodology was subsequently employed by other investigators in studies of *Borrelia* viability [29,30,31]. 

Heat-inactivated sera (45 min at 56 °C) of 1 male and 1 female from group 3 (41–50 years old) were used as negative controls. Fifty microliters of *Borrelia* culture, for each species, with cell density 10^7^ cells/mL, was added in a ratio of 1:1 to individual serum samples and co-incubated at 34 °C for 24 h in microtiter plates. Samples were then supplemented with 100 µL of dilution buffer (2% BSA, 5.4 mM glucose in sterile PBS) and transferred to 5 mL BD Falcon tubes. Then, 1 µL of propidium-iodide (final concentration 2 µg/mL) was added, and reactions were incubated in the dark for 15 min after the addition of propidium-iodide. Flow cytometric analysis of 30,000 events per sample was performed using a BD FACSCanto II cytometer and FACSDiva software (v. 5.0, BD Biosciences, San Jose, CA, USA). Single *Borrelia* cells were isolated, and cell aggregates or debris were digitally removed, using sequential FSC-A/SSC-A, FSC-H/FSC-A, and SSC-W/SSC-H gating strategies. Propidium iodide fluorescence intensity was quantified exclusively within this gated singlet population using the PE-Texas Red-A channel (616/23 nm). The resulting values indicate the percentage of dead *Borrelia* in the individual samples. The average percentage of dead *Borrelia* in each group was calculated from 10 measurements. Each time the assay was run, a live spirochete control (the same spirochete species in BSK-H medium) was used. Propidium-iodide positivity was defined using heat-killed *Borrelia* control (the same spirochete culture heated at 56 °C for 45 min), and the threshold was positioned at the fluorescence intensity separating the negative and positive populations. Because propidium-iodide was the only fluorochrome used, compensation was not applied. While propidium iodide is a highly effective indicator of membrane damage, relying on it as a standalone metric for complement-mediated killing introduces biological and technical limitations. The use of flow cytometry and sequential gating detects only late-stage membrane permeabilization and would exclude cells that were showing modest damage, for example, “blebbing” and altered motility, as shown in other studies assessing *Borrelia* cell viability by microscopy. Similarly aggregates and debris that might be counted microscopically were removed from the flow cytometry counts. Despite this constraint, flow cytometry serves as a high-throughput surrogate for terminal complement-mediated lysis. Ultimately, the fluorescence enhancement of propidium-iodide upon nucleic acid binding provides a binary readout of irreversible outer and inner membrane disruption caused by the membrane attack complex. In total, 1200 complement sensitivity tests were performed, and all samples and controls were analyzed in the same way. Supplemental Table S1 provides the data from 10 measurements for each sample.

### 2.4. Statistical Analysis

The survival of *Borrelia* species (Supplemental Table S1) was first compared using a Kruskal–Wallis test, followed by Dunn’s post hoc pairwise comparisons (Supplemental Table S2), to categorize them into low-, medium-, and high-sensitivity groups based on significantly different levels of complement-mediated killing. Once divided into sensitivity groups, the effects of age, biological sex, and their interaction were assessed using an Aligned Rank Transform (ART) ANOVA (Supplemental Table S3). Where significant interactions were detected, post hoc Wilcoxon rank-sum tests were conducted within each age category to compare males and females separately, allowing assessment of sex-specific effects within each age category. All post hoc comparisons were corrected for multiple testing using the Holm–Bonferroni method. Patterns across age and sex combinations were visualized using Principal Coordinate Analysis (PCoA) based on Bray–Curtis distances.

## 3. Results

### 3.1. Human Host Complement-Mediated Borrelia Mortality Is Dependent on Borrelia Species

**Figure 1:**
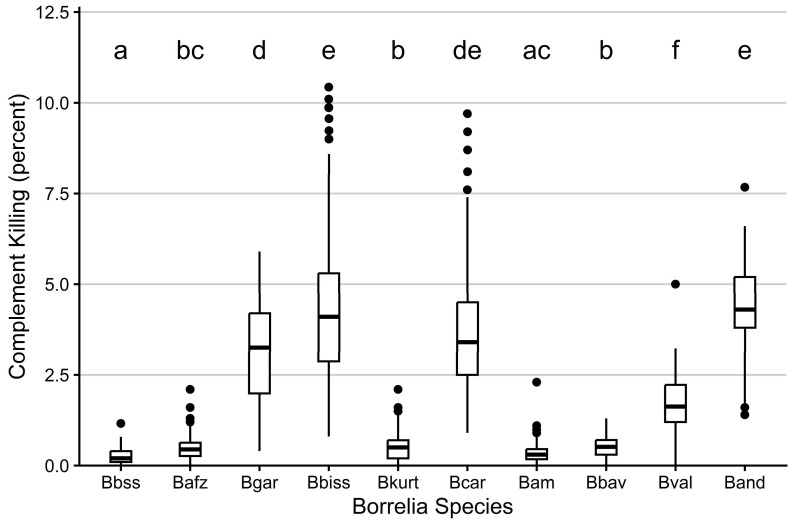
Complement-mediated killing of 10 different *Borrelia* species. The percent complement-mediated killing of different *Borrelia* species for combined age and donor sex data was used to define low-, medium- and high-sensitivity groups. Box plots sharing letters are not significantly different in pairwise comparisons. For example, Bbss (a) is significantly different from Bafz (b, c) but is not significantly different from Bam (a, c). Bval (f) is significantly different from all of the other species. Bbss = *B. burgdorferi* sensu stricto, Bafz = *B. afzelii*, Bgar = *B. garinii*, Bbiss = *B. bissettiae*, Bkurt = *B. kurtenbachii*, Bcar = *B. carolinensis*, Bam = *B. americana*, Bbav *= B. bavariensis*, Bval = *B. valaisiana*, Band = *B. andersonii*.

The human complement system is an essential part of innate immunity and has antibacterial properties. Examining *Borrelia* cell viability after exposing ten different spirochete species to human complement indicates whether the complement is protective against that *Borrelia* species. Evaluation of the results of host complement killing of the different *Borrelia* species was assessed using the Kruskal–Wallis H test, using the combined age and gender data for the complement-mediated *Borrelia* killing for each *Borrelia* species (Figure 1, Supplemental Table S1). A Kruskal–Wallis rank-sum test showed a significant difference in complement-mediated killing among the *Borrelia* species (χ^2^(9) = 914.12, *p* < 2.2 × 10^−16^), indicating a highly statistically significant difference in serum sensitivity between the species. The Dunn test was used to do post hoc pairwise comparisons. Of the 45 comparisons, only nine were not significant (Bafz—Bam, Bafz—Bbav, Band—Bbiss, Bam—Bbss, Band—Bcar, Bbiss—Bcar, Bafz—Bkurt, Bcar—Bgar, Bbav—Bkurt; Figure 1).

These findings delineated three groups with statistically significant differences in their serum sensitivity (Figure 1).

The group with the lowest complement-mediated killing in response to challenge by human complement included *B. burgdorferi* sensu stricto (Bbss, a), *B. afzelii* (Bafz, bc), *B. kurtenbachii* (Bkurt, b), *B. americana* (Bam, ac), and *B. bavariensis* (Bbav, b); *B. burgdorferi* s.s. and *B. americana* showed the highest serum resistance within this group. *Borrelia afzelii*, *B. bavariensis* and *B. kurtenbachii* shared low serum sensitivity under these experimental conditions. The serum sensitivity of *B. valaisiana* (f) was intermediate. The group with the highest sensitivity to the human serum included *B. garinii*, *B. bissettiae*, *B. carolinensis* and *B. andersonii* (which was the species most sensitive to human serum) (Figure 1). The serum sensitivities of the tested strains of *B. bissettiae* and *B. carolinensis* were comparable to that of *B. garinii* under these ex vivo experimental conditions. Our results are consistent with the earlier published findings of the same gradient of serum sensitivity of multiple strains of different *Borrelia* species [8,17,27,28].

### 3.2. Effect of Biological Sex and Age on Complement-Mediated Borrelia Killing

To test the effect of age and sex on complement-mediated *Borrelia* killing, it was first necessary to assess whether there was an interaction between age and sex to determine if the results could be readily interpreted. If there was no interaction, the main effects could be interpreted directly; however, if an interaction were present, the results could not be interpreted without further analysis and consideration of limitations. Aligned Rank Transform ANOVA tests (Supplementary Table S3) demonstrated that the low- (Bbss (a), Bafz (bc), Bkurt (b), Bam (ac), Bbav (b) and medium-serum-sensitivity (Bval) groups showed no significant interaction between age and sex of the host (*p* = 0.6334 for the low-sensitivity group and *p* = 0.1346 for the medium-sensitivity group). However, the high-sensitivity group (Bgar (d), Bbiss (e), Bcar (de), Band(e)) showed a significant interaction (*p* = 0.0008), so the effects of age or sex cannot be interpreted independently.

**Figure 2:**
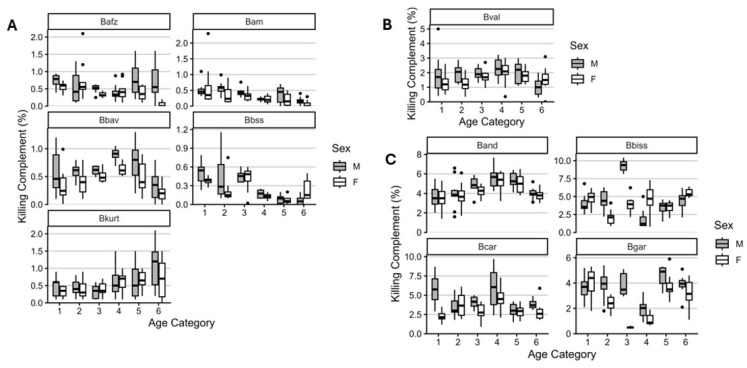
The effect of host biological sex and age on complement-mediated killing of different *Borrelia* species. (**A**) The percent of complement-mediated *Borrelia* killing for the low-sensitivity *Borrelia* species. (**B**) Percent of complement-mediated *Borrelia* killing for the medium-sensitivity *Borrelia* species. (**C**) Percent of complement-mediated *Borrelia* killing for the high-sensitivity *Borrelia* species. Age categories 1–6 are 1 = 21–30 years, 2 = 31–40 years, 3 = 41–50 years, 4 = 51–60 years, 5 = 61–70 years, and 6 = 70+ years, with host biological sex indicated by box shading (filled = male, unfilled = female). Bbss = *B. burgdorferi* sensu stricto, Bafz = *B. afzelii*, Bgar = *B. garinii*, Bbiss = *B. bissettiae*, Bkurt = *B. kurtenbachii*, Bcar = *B. carolinensis*, Bam = *B. americana*, Bbav = *B. bavariensis*, Bval = *B. valaisiana*, Band = *B. andersonii*.

Biological sex consistently affected complement-mediated *Borrelia* killing, with females showing reduced killing compared to males across nearly all age groups and *Borrelia* species (Figure 2A–C). For the low-sensitivity group, the decreased *Borrelia* killing in females was statistically significant (*p* = 2.62 × 10^−6^). For the single medium-sensitivity species, the effect was not statistically significant (*p* = 0.1158), although visual examination of Figure 2B shows decreased complement-mediated *Borrelia* killing in females in several age groups. Although the interaction between age and sex complicates the interpretation of biological sex’s effect on complement-mediated *Borrelia* killing in the high-sensitivity group, this effect remains evident.

For the low- and medium-serum-sensitivity groups, there is a significant effect of age on complement-mediated *Borrelia* killing (*p* = 1.02 × 10^−5^ and *p* = 0.0018, respectively; Figure 2A,B). For *B. burgdorferi* s.s., *B. afzelii* and *B. americana*, the serum sensitivity generally declined with age. Conversely, *B. kurtenbachii* exhibits the opposite trend, indicating a possible increase in complement-mediated killing in older age groups. For *B. bavariensis* and *B. valaisiana*, a non-linear pattern appears to emerge, where the highest complement-mediated *Borrelia* killing is observed within the 51–60 age group, while younger and older individuals exhibit reduced killing efficiency. A similar trend extends to other highly sensitive *Borrelia* species (Figure 2C), though an interaction between age and sex introduces additional complexity to the interpretation of the findings.

**Figure 3:**
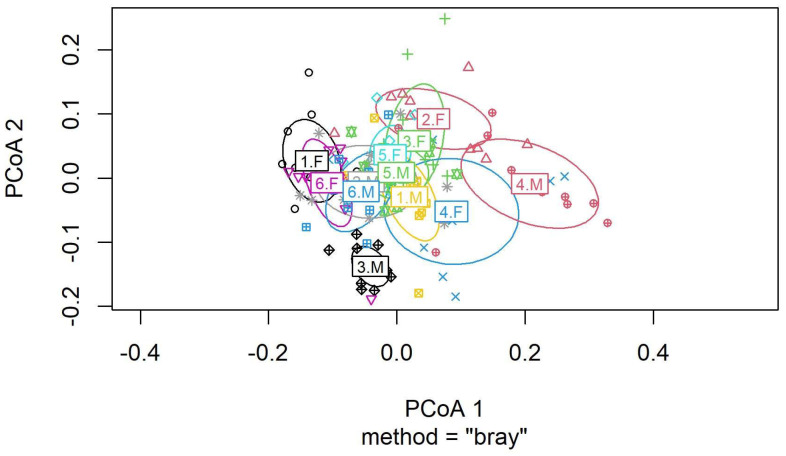
Principal Coordinate Analysis of complement-mediated *Borrelia* killing across all *Borrelia* species, based on Bray–Curtis distances. The plot illustrates relative dissimilarities among all age–sex combinations, with the 3M group appearing as the most distinct.

Pairwise post hoc comparisons, with age and sex combined into groups, using the Wilcoxon rank-sum test with continuity correction (Supplemental Table S4), showed *p* values below 0.05 for males aged 41–50 years (group 3M). This is illustrated with a Principal Coordinate Analysis (Figure 3) showing the relationships among different age groups across all *Borrelia* species based on Bray–Curtis distances. The group of 41- to 50-year-old males (3M) is the most dissimilar, highlighting a possible synergistic effect of age and gender on complement-mediated *Borrelia* killing in this group.

## 4. Discussion

The findings of this study have several important implications. We found that resistance to human serum was similar between some well-known pathogenic species recognized as the major etiological agents of Lyme borreliosis worldwide and those less frequently reported in humans or with unclear pathogenicity toward humans. 

The absolute percentage of complement-mediated cell death is not directly comparable between studies using different methods to assess *Borrelia* viability, however, the relative viability is comparable. For example, studies utilizing immobilization and/or dark-field microscopy and/or flow cytometry will produce different measures of serum susceptibility depending on the specific conditions of the assay [32,33,34]; however, the relative effects of complement activity are consistent across individual studies and between studies, with the caveat that sensitivity depends on the *Borrelia* strain. For example, Bhide et al. (2005) [34] reported serum sensitivities ranging from 41% to 92% for *B.* garinii (although some of this range may have been due strains later reclassified as *Borrelia bavariensis*). Importantly, despite differences in the absolute levels of serum sensitivity, the relative ranking of species closely matched that observed in the present study, with the exception of *B. bissettii*. In both studies, the species followed the same overall order of increasing serum sensitivity, from *B. burgdorferi* sensu stricto through *B. afzelii, B. valaisiana,* and *B. andersonii* to *B. garinii*, whereas *B. bissettii* occupied a different relative position. Similarly, van Dam et al. [32] reported serum sensitivities ranging from 5% to 51% for *B. burgdorferi*, depending on the strain examined. Although the absolute values differed among studies, the relative difference between *B. garinii* and *B. burgdorferi* was remarkably consistent. Bhide et al. [34] found *B. garinii* to be approximately 5-fold more serum-sensitive than *B. burgdorferi*, while van Dam et al. [32] and Breitner-Ruddock et al. [33] reported differences ranging from approximately 2- to 20-fold and 5- to 20-fold, respectively. In the present study, *B. garinii* exhibited approximately 9-fold greater serum susceptibility than *B. burgdorferi*. Thus, while the average serum susceptibility rate for all *Borrelia* species under these experimental conditions remained below 10%, the relative effect of human complement on *Borrelia* cell permeabilization was consistent with prior studies. Although flow cytometry is less suited than imaging-based approaches for detecting subtle changes in spirochete morphology and behavior, its high throughput enables rapid, standardized analysis of large numbers of samples. This facilitated the inclusion of sufficient biological replicates for statistical comparisons of the relative susceptibility of *Borrelia* species to human complement, in particular with respect to biological sex and age. The resulting dataset revealed a consistent difference in complement-mediated killing between sera from female and male donors, with reduced killing observed in female sera across nearly all age groups and *Borrelia* species. Although the mechanisms underlying this observation remain unknown, its reproducibility across independent samples suggests that it represents a genuine biological phenomenon worthy of further investigation. Future time-resolved studies combining flow cytometry with high-resolution imaging could further characterize changes in *Borrelia* morphology and behavior during exposure to human complement and help elucidate the mechanisms underlying the observed sex-specific differences in complement-mediated killing.

The finding that *Borrelia garinii* clustered among the most serum-sensitive species despite being one of the major confirmed human pathogens also deserves attention. Rather than representing a contradiction, this observation highlights an important biological principle: although complement-mediated killing represents a vital barrier to infection, resistance to human complement alone cannot fully explain pathogenicity in Lyme borreliosis spirochetes. Multiple factors influence the complex interplay between a pathogen, vector and an individual host. This study focuses on the role of the human complement in repelling infection, and our results show that the human complement alone may not serve as a reliable barrier to infection by *Borrelia* species should humans be exposed to them. For example, the resistance of *B. americana* to human serum was the same as that of *B. burgdorferi* s.s., the major cause of LB in North America, and complement-mediated killing was not statistically different between *B. kurtenbachii* and pathogenic Eurasian species *B. afzelii* and *B. bavariensis*. Likewise, *B. bissettiae* and *B. carolinensis* have a sensitivity to complement-mediated killing similar to that of *B. garinii*, the second most important cause of human LB in Europe [35,36,37]. The sensitivity of *B. garinii* and *B. bavariensis* to human serum complement differs, as is also the case for *B. bissettiae* and *B. kurtenbachii*, reinforcing the biological distinction between these species.

The first indication of this host-pathogen complexity emerged from the seminal work of van Dam and colleagues, who demonstrated that many *B. garinii* isolates were highly susceptible to normal human serum in vitro [32]. Under exposure to normal human serum, susceptible isolates progressively lost motility, developed membrane alterations, and were ultimately eliminated, demonstrating efficient complement-mediated killing. Surprisingly, four of the serum-sensitive isolates had been recovered from the cerebrospinal fluid of patients with neuroborreliosis, providing unequivocal evidence that dissemination into humans can occur despite pronounced complement susceptibility [32]. This observation challenged the prevailing view that invasive disease is restricted to serum-resistant spirochetes, suggesting that complement resistance modifies the probability and efficiency of successful infection rather than acting as an all-or-none determinant of virulence.

Subsequent studies revealed that the apparent paradox is further complicated by the biological heterogeneity within the traditional *B. garinii* group. While OspA serotypes 5 and 6 isolates were predominantly serum sensitive, van Dam et al. observed that most OspA serotype 4 isolates exhibited substantial serum resistance [9,28]. Mechanistic investigations subsequently demonstrated that the neuroinvasive strain PBi, originally classified as *B. garinii* OspA serotype 4, survived exposure to 50% normal human serum at levels comparable to *B. burgdorferi* sensu stricto B31 and recruited the complement regulator factor H-like protein 1 (FHL-1) through the surface proteins BGA66 and BGA71 [38]. Further work showed that these proteins inhibit assembly of the membrane attack complex, thereby conferring effective complement resistance and function as direct terminal pathway inhibitors by physically interacting with late complement components C7, C8, and C9 [39,40]. This interaction blocks C9 polymerization, thereby halting the assembly of the pore-forming membrane attack complex (MAC) and facilitating spirochetal immune evasion [39]. Importantly, serum-resistant strain PBi was subsequently recognized as the distinct spirochete species *B. bavariensis* [41]. However, reclassification does not fully resolve the issue. Even after the separation of *B. bavariensis*, serum-sensitive *B. garinii* isolates have continued to be associated with human disease, including neuroborreliosis [42]. Thus, the ability to invade the human host and the ability to withstand prolonged exposure to circulating complement cannot be regarded as strictly equivalent traits. Instead, the persistence of this phenomenon suggests that complement susceptibility and pathogenic potential are linked in a more complex and context-dependent manner.

Complement resistance represents only one component of a multifactorial virulence strategy that operates dynamically throughout the enzootic cycle. During natural transmission, spirochetes encounter a succession of immunologically distinct environments, including the tick midgut, tick saliva, the skin at the inoculation site, blood, lymphatic tissues, and ultimately target organs. Expression of complement-evasion molecules is tightly regulated by environmental cues and differs substantially between in vitro cultivation and mammalian infection. Consequently, serum susceptibility assays performed on cultured organisms may not accurately reflect the phenotype expressed during tick-to-host transmission. Rather than requiring constitutive resistance throughout infection, spirochetes may only need a transient period of complement protection sufficient to survive initial dissemination before establishing themselves within tissues where complement pressure is diminished. This interpretation is supported by experimental evidence indicating that individual CRASP family members are differentially deployed during distinct phases of the tick–vertebrate cycle of complement regulator-acquiring surface proteins (CRASPs), indicating that complement evasion is a dynamic and adaptable process rather than a fixed characteristic of a given *Borrelia* species [20,43].

This concept may be particularly relevant for neurotropic *Borrelia*. Compared with plasma, CSF contains substantially lower concentrations of complement proteins and exhibits reduced terminal pathway activity. Species or strains with limited capacity to withstand extended exposure to circulating complement may therefore still establish infection if they rapidly access anatomical niches characterized by diminished complement-mediated antimicrobial pressure. The well-recognized association of *B. garinii* and *B. bavariensis* with neuroborreliosis is consistent with this hypothesis and raises the possibility that neurotropism itself may reduce the selective advantage conferred by constitutive serum resistance. More broadly, observations across LB spirochetes indicate that complement resistance alone cannot account for pathogenic potential. Human-pathogenic species differ considerably in tissue tropism and clinical manifestations despite sharing many complement-evasion strategies. Conversely, serum-resistant phenotypes occur among species exhibiting different capacities for dissemination and host association. Beyond surviving complement attacks, the ultimate virulence of *Borrelia* depends on multiple strategies including antigenic variation and protective tick salivary proteins to dictate the ultimate clinical trajectory of the infection [16,43].

Taken together, this results of this study support a more nuanced correlation between complement resistance and pathogenicity; a binary interpretation of complement resistance as either present or absent correlating directly with pathogenic or non-pathogenic is not supported. Instead, our findings support an emerging model in which complement-mediated selection may shape host associations and transmission success but without strictly dictating pathogenic outcome or serving as an absolute predictor of virulence [44]. Human pathogenicity in LB spirochetes appears to arise from the interplay between complement susceptibility, inducible immune-evasion mechanisms, strain-specific heterogeneity, tissue tropism, and additional virulence determinants. In this context, the relationship between complement resistance and pathogenicity is quantitative, dynamic, and context-dependent. Appreciating this complexity may ultimately provide a more accurate framework for understanding host adaptation, tissue tropism, and the evolution of virulence within the *Borrelia burgdorferi* sensu lato complex.

## Data Availability

The original contributions presented in this study are included in the article/Supplementary Material. Further inquiries can be directed to the corresponding author.

## Supplementary Materials

The following supporting information can be downloaded at: https://www.mdpi.com/article/10.3390/pathogens15070717/s1; Supplementary Table S1: Raw complement-mediated killing (%) of 10 *Borrelia* species by human sera, separated by age category and biological sex; Supplementary Table S2: Kruskal–Wallis rank-sum test and post hoc Dunn’s test results comparing complement-mediated killing across 10 *Borrelia* species; Supplementary Table S3: Results of the ART ANOVAs, testing the effect of age category and sex on complement-mediated killing; Supplementary Table S4: Wilcoxon rank-sum test results comparing complement-mediated killing between sexes within each age category for high-, medium-, and low-sensitivity *Borrelia* groups.
