# Supplementary material for: Human Complement Evasion Is Widespread Among Lyme Borreliosis Spirochete Species
Source: Pathogens. 2026 Jul 7;15(7):717. doi: 10.3390/pathogens15070717 (PMC13414706; doi:10.3390/pathogens15070717)
Supplement: Supplementary file 1 [file pathogens-15-00717-s001.zip › pathogens-4357317-supplementary.pdf]

Supplementary Table S1. Raw complement-mediated killing (%) of 10 *Borrelia* species by human sera, separated by age category and biological sex.

Note: Bbss = *B. burgdorferi sensu stricto*; Bafz = *B. afzelii*; Bgar = *B. garinii*; Bbis = *B. bissettiae*; Bkurt = *B. kurtenbachii*; Bcar = *B. carolinensis*; Bam = *B. americana*; Bbav = *B. bavvariensis*; Bval = *B. valaisiana*; Band = *B. andersonii*. Values represent percentage mortality. Data are stratified by age category (1 (21-30), 2 (31-40), 3 (41-50), 4 (51-60), 5 (61-70), 6 (70+) and sex (F/M).

	Bbss		Bafz		Bgar		Bbis		Bkurt		Bcar		Bam		Bbav		Bval		Band	
	F	M	F	M	F	M	F	M	F	M	F	M	F	M	F	M	F	M	F	M
Cat 1 (21-30 y)	0.27	0.23	0.61	0.74	4.6	3.8	5.5	3.5	0.5	0.6	2.3	5.3	0.3	0.6	0.5	0.55	1.5	1.6	3.7	3.8
	0.35	0.26	0.63	0.89	5.2	4.2	4.1	2.8	0.2	0.6	2.1	6.2	0.7	0.32	0.1	0.16	1.1	1.1	3.1	3.2
	0.39	0.6	0.74	0.94	3.9	3	3.8	4.5	0.2	0.1	2.2	4.8	0.5	0.49	0.55	0.28	0.8	2	2.9	5.5
	0.34	0.51	0.51	0.42	4.2	2.8	2.7	5.1	0.1	0.7	2	2.9	0.2	1.1	0.4	1.2	0.5	5	2.5	2.9
	0.47	0.59	0.47	0.39	4.8	2.1	5	3.6	0.5	0.2	1.9	3.4	2.3	0.42	0.21	0.1	2.1	0.9	5.3	2.9
	0.38	0.58	0.42	0.61	5.3	5.2	6.2	2.5	0.1	0.2	2.7	7.2	0.2	0.39	0.01	0.37	0.9	1.8	4.2	2
	0.45	0.49	0.58	0.87	3.2	3.3	5.8	4.2	0.4	0.6	1.2	4.3	0.4	0.5	0.28	0.74	1.3	2.3	3.3	2.8
	0.39	0.61	0.7	0.71	1.8	3.6	5.3	3.3	0.5	0.6	3.5	6.9	0.2	0.4	0.99	0.37	2.6	0.7	1.4	5.2
	0.35	0.79	0.31	0.9	4.9	3.8	4.9	6.8	0.4	0.9	1.2	8.7	1.1	0.33	0.16	0.99	1.6	2.9	4.2	4.6
	0.41	0.34	0.63	0.83	3.1	4.2	4.7	3.5	0.3	0.2	3.2	7.6	0.3	0.55	0.2	0.94	0.6	0.4	4.5	3.8
total	3.8	5	5.6	7.3	41	36	48	39.8	3.2	4.7	22.3	57.3	6.2	5.1	3.4	5.7	13	18.7	35.1	36.7
**average**	**0.38**	**0.5**	**0.56**	**0.73**	**4.1**	**3.6**	**4.8**	**3.98**	**0.32**	**0.47**	**2.23**	**5.73**	**0.62**	**0.51**	**0.34**	**0.57**	**1.3**	**1.87**	**3.51**	**3.67**
Cat 2 (31-40y)	0.11	0.33	0.69	0.62	2.6	4.1	2.3	3.8	0.4	0.6	3.4	3.1	0.28	0.46	0.3	0.63	1.26	2.88	3.4	3.8
	0.2	0.24	0.23	0.43	3.1	3.6	1.9	2.2	0.9	0.3	3.9	2.8	0.19	0.58	0.1	0.62	0.35	1.25	3.2	4.4
	0.3	0.15	0.55	0.23	2.1	4.4	3.2	5.3	0.1	0.4	2.3	4.5	0.29	0.25	0.5	0.42	0.95	2.35	4.3	3.8
	0.15	0.67	0.47	0.1	1.8	1.8	1	4.9	0.2	0.2	1.9	2.3	0.9	0.72	0.2	0.72	1.56	1.28	5.1	2.1
	0.13	0.55	2.1	1.2	2	4.5	2.4	3	0.1	0.8	5.5	4.1	0.8	0.59	0.6	0.34	1.58	1.91	2.9	1.6
	0.2	0.68	0.21	0.98	2.8	2.2	0.9	3.8	0.2	0.1	6.1	4.3	0.6	0.29	0.4	0.61	1.1	1.55	3.3	6.2
	0.75	0.22	0.53	1.3	2.9	5.4	2.3	5.8	0.4	0.3	1.9	2.9	0.12	0.63	0.8	0.58	2.2	1.18	4.1	3.9
	0.1	1.16	0.56	0.4	3.1	3.8	4.1	3.9	0.7	0.6	2.7	5.7	0.18	0.5	0.5	0.5	1.9	2.3	1.7	6.6

	0.15	0.1	1.2	0.13	2.2	3.5	1.8	6.3	0.2	0.4	5.3	2.8	0.13	0.59	0.4	0.68	0.9	2.7	3.9	4.01
	0.12	0.1	0.66	0.11	1.4	5	1.1	5.9	0.6	0.6	4	1.8	0.11	0.99	0.2	0.8	0.7	2.2	6.1	3.69
total	2.21	4.20	7.20	5.50	24.00	38.30	21.00	44.90	3.80	4.30	37.00	34.30	3.60	5.60	4.00	5.90	12.50	19.60	38.00	40.10
**average**	**0.21**	**0.42**	**0.72**	**0.55**	**2.4**	**3.83**	**2.1**	**4.49**	**0.38**	**0.43**	**3.7**	**3.43**	**0.36**	**0.56**	**0.4**	**0.59**	**1.25**	**1.96**	**3.8**	**4.01**
Cat 3 (41-50)	0.59	0.49	0.39	0.53	0.63	3.31	3.65	9	0.6	0.1	2.8	4.9	0.2	0.36	0.41	0.61	1.75	1.9	4.01	4.5
	0.51	0.5	0.45	0.61	0.46	3.57	4.01	8.21	0.3	0.2	3.5	3.2	0.39	0.45	0.45	0.7	1.89	2.36	4.67	4.69
	0.6	0.61	0.31	0.6	0.48	4.25	4.59	8.59	0.6	0.4	4.2	3.8	0.35	0.39	0.39	0.49	1.23	2.54	3.87	3.09
	0.02	0.35	0.29	0.48	0.54	4.97	6.23	9.23	0.3	0.1	2.7	4.8	0.4	0.5	0.51	0.52	1.62	1.87	3.61	4.5
	0.46	0.36	0.23	0.39	0.57	4.91	4.56	9.56	0.4	0.5	2.3	5	0.15	0.41	0.58	0.63	1.59	1.63	4.53	4.98
	0.42	0.42	0.4	0.52	0.54	3.07	3.95	9.87	0.1	0.1	3.8	2.9	0.64	0.35	0.45	0.69	1.48	1.3	4.91	5.65
	0.51	0.35	0.27	0.63	0.43	3.39	3.91	8.15	0.4	0.3	4	3.5	0.3	0.27	0.52	0.54	1.98	2.01	5.01	5.68
	0.54	0.49	0.37	0.25	0.4	2.95	3.21	9.86	0.7	0.6	2.5	4.6	0.38	0.48	0.58	0.56	2.01	2.6	4.6	4.72
	0.36	0.36	0.3	0.56	0.5	2.98	2.75	10.1	0.1	0.4	2.3	4.3	0.19	0.43	0.39	0.69	0.95	1.89	3.9	4.99
	0.19	0.57	0.32	0.53	0.45	5.1	2.14	10.43	0.3	0.5	0.9	4	0.2	0.76	0.72	0.67	2.7	1.4	3.19	5.9
total	4.2	4.5	3.33	5.1	5	38.5	39	93	3.8	3.2	29	41	3.2	4.4	5	6.1	17.2	19.5	42.3	48.7
**average**	**0.42**	**0.45**	**0.33**	**0.51**	**0.5**	**3.85**	**3.9**	**9.3**	**0.38**	**0.32**	**2.9**	**4.1**	**0.32**	**0.44**	**0.5**	**0.61**	**1.72**	**1.95**	**4.23**	**4.87**
Cat 4 (51-60)	0.09	0.15	0.47	0.3	0.9	1.82	4.5	1.4	0.7	0.8	4.3	7.4	0.19	0.2	0.76	0.94	2.9	3.23	6.24	6.02
	0.2	0.11	0.35	0.45	1.6	2.1	4.4	0.9	0.4	0.1	3.8	6.2	0.16	0.31	0.81	0.98	2.36	3.01	3.21	6.15
	0.14	0.23	0.92	0.38	1.8	1.96	3.6	1.1	0.8	0.4	2.5	2.9	0.35	0.19	0.54	0.74	3.01	2.95	5.59	5.26
	0.1	0.09	0.25	0.28	0.85	1.32	1.8	2.4	0.3	1.4	6.1	3.3	0.35	0.25	0.51	0.69	1.96	2.64	5.41	5.89
	0.11	0.25	0.1	0.33	0.66	0.9	7.1	0.9	0.1	0.3	4.7	8.1	0.2	0.21	0.63	0.84	1.84	1.56	6.23	5.47
	0.12	0.23	0.44	0.61	0.84	2.3	0.8	1.2	1	0.2	5.5	5.9	0.2	0.3	0.72	0.86	2.54	1.23	4.75	4.59
	0.15	0.1	0.37	0.12	0.99	3.3	7.3	3	0.6	0.8	7.3	2.4	0.06	0.18	0.55	0.93	2.19	1.98	4.9	4.6
	0.18	0.24	0.51	0.88	0.68	2.6	4.9	5	1	0.6	4.5	9.7	0.4	0.12	0.59	1.05	1.98	1.79	6.3	4.23
	0.05	0.1	0.12	0.12	0.78	1.1	6.1	0.9	0.7	0.4	4.5	9.2	0.12	0.23	0.67	0.89	0.96	2.01	5.4	6.12
	0.16	0.2	0.87	0.33	1.9	2.3	5.5	1.2	0.7	1.5	2.1	5.2	0.07	0.21	0.52	0.98	0.36	2.5	3.97	7.67
total	1.30	1.70	4.40	3.80	11.00	19.70	46.00	18.00	6.30	6.50	45.30	60.30	2.10	2.20	6.30	8.90	20.10	22.90	52.00	56.00
**average**	**0.13**	**0.17**	**0.44**	**0.38**	**1.1**	**1.97**	**4.6**	**1.8**	**0.63**	**0.65**	**4.53**	**6.03**	**0.21**	**0.22**	**0.63**	**0.89**	**2.01**	**2.29**	**5.2**	**5.6**

Cat 5 (61-70)	0.01	0.01	0.1	0.8	4.1	5.2	3.6	4.1	0.4	0.2	3.1	4.2	0.1	0	0.1	0.7	1.8	2.1	5	5
	0.1	0.05	0.2	0.4	4.5	5	4.1	4.6	0.9	0.6	3.5	3.9	0	0.1	0.5	0.2	2.1	2.3	5.2	5.2
	0.09	0.09	0.6	0.2	2.9	5.3	4.2	3.9	0.8	1	2.8	1.5	0	0.2	0.8	1.3	2.3	1.2	5.9	6.2
	0.2	0.01	0.4	0.3	2.5	4.8	4.5	3.2	1	1.1	2.5	2.6	0	0.7	0.2	0.5	1.3	1.1	6	4.1
	0.1	0.01	0.3	0.6	3.4	4.2	4.1	3.8	0.2	1.5	4.2	2.8	0.2	0.4	0.8	0.9	1.2	2.6	4.2	5.3
	0.02	0.2	0.1	1.6	3.3	3.9	3.2	3.7	0.9	0.4	3.4	2.3	0.3	0.5	0.3	0.6	2	1.4	3.8	5.6
	0.03	0.1	0.54	0.4	3.7	3.8	2.4	4.2	0.6	0.2	1.6	4.1	0.4	0.1	0.5	0.5	1.8	1.1	4.1	4.8
	0.06	0.2	0.63	1.3	3.6	3.2	2	2.9	0.7	0.9	2.3	2.5	0.5	0.5	0.9	1	1.4	2.5	5	6.4
	0.05	0.13	0.24	1.2	3.4	5.2	3	1.5	0.6	0.1	2.1	3.5	0.4	0.6	0.3	1.2	2.6	3	6.5	6.3
	0.04	0.1	0.59	0.8	5.9	5.2	3.9	1.6	0.5	0.1	3.1	3.2	0.1	0.6	0.3	0.9	1.4	2.6	4	4.9
total	0.7	0.9	3.7	7.6	37.3	45.8	35	33.5	6.6	6.1	28.6	30.6	2	3.7	4.7	7.8	17.9	19.9	49.7	53.8
**average**	**0.07**	**0.09**	**0.37**	**0.76**	**3.73**	**4.58**	**3.5**	**3.35**	**0.66**	**0.61**	**2.86**	**3.06**	**0.2**	**0.37**	**0.47**	**0.78**	**1.79**	**1.99**	**4.97**	**5.38**
Cat 6 (>71)	0.1	0	0	0	1.1	4	5.2	5.2	0	1.2	3	4	0	0.2	0.2	0.3	1.5	1.1	3	4
	0.3	0	0.1	0.4	3.1	4.2	5.3	3.5	1	0.5	3.2	4.2	0	0.3	0.3	0.1	2	0.3	3.2	3.8
	0.1	0	0.2	0.1	3.2	5.1	6.2	6.2	1.2	0.6	3.4	3.4	0.1	0.1	0.2	0.5	2.3	0.5	3.6	3.9
	0.4	0.1	0.1	0.6	3.1	3.9	4.8	2.4	1.4	0.3	2.1	3.2	0.2	0.2	0.1	0.8	3.1	1.6	3.4	3.2
	0.5	0.1	0	1.1	2	3.7	5	4.5	1.5	0.2	2.2	3.1	0.3	0	0.5	0.7	1.2	1.5	3.8	3.1
	0.4	0.2	0	1.2	4.6	4.2	5.1	4.8	0	1.2	1.9	3.4	0	0.1	0.2	0.2	1.5	0.4	4	4.2
	0.1	0.1	0	0.4	2.5	3.5	6.1	4.9	0.1	2.1	1.8	4.1	0	0	0.1	0.5	1.6	0.6	3.8	4.5
	0.1	0	0	0.5	3.6	3.7	5.6	2.1	0.8	1.4	2	3.4	0	0.4	0	0	1.2	0.9	4.1	5.2
	0.2	0	0.1	0.9	4.2	2.1	5.3	4.6	0.4	1.6	3.1	4.7	0.1	0.2	0.4	0	1.1	1.2	4.2	4.3
	0.1	0.1	0	1.6	4.6	4.4	6.4	5.8	0.6	1.5	5.9	4.9	0	0.1	0.1	0.4	0	2	4.9	3.9
total	2.3	0.6	0.5	6.8	32	38.8	55	44	7	10.6	28.6	38.4	0.7	1.6	2.1	3.5	15.5	10.1	38	40.1
**average**	**0.23**	**0.06**	**0.5**	**0.68**	**3.2**	**3.88**	**5.5**	**4.4**	**0.7**	**1.06**	**2.86**	**3.84**	**0.07**	**0.16**	**0.21**	**0.35**	**1.55**	**1.01**	**3.8**	**4.01**

Supplementary Table S2. Kruskal-Wallis rank sum test and post-hoc Dunn's test results comparing complement-mediated killing across 10 *Borrelia* species. P-values adjusted using the Holm-Bonferroni method. Significance levels: * $p \leq 0.05$, ** $p \leq 0.01$, *** $p \leq 0.001$. df = degrees of freedom.

Kruskal-Wallis rank sum test:

$\chi^2 = 914.12$, df = 9, $p < 2.2 \times 10^{-16}$

Dunn's test:

Comparison	Z score	Raw *p*-value	Adjusted *p*-value
Bafz - Bam	2.27	0.0115	0.104
Bafz - Band	-14.72	2.35×10^{-49}	9.16×10^{-48} ***
Bam - Band	-16.99	4.55×10^{-65}	2×10^{-63} ***
Bafz - Bbav	-0.64	0.26	0.78
Bam - Bbav	-2.92	0.00177	0.0213*
Band - Bbav	14.08	2.59×10^{-45}	9.32×10^{-44} ***
Bafz - Bbis	-13.68	6.45×10^{-43}	2.26×10^{-41} ***
Bam - Bbis	-15.96	1.31×10^{-57}	5.37×10^{-56} ***
Band - Bbis	1.04	0.149	0.897
Bbav - Bbis	-13.04	3.65×10^{-39}	1.17×10^{-37} ***
Bafz - Bbss	3.28	0.000511	0.00664 **
Bam - Bbss	1.01	0.156	0.779
Band - Bbss	18.01	8.76×10^{-73}	3.94×10^{-71} ***
Bbav - Bbss	3.93	4.29×10^{-5}	0.000729 ***
Bbis - Bbss	16.97	7.2×10^{-65}	3.09×10^{-63} ***
Bafz - Bcar	-12.78	1×10^{-37}	3.11×10^{-36} ***
Bam - Bcar	-15.06	1.56×10^{-51}	6.22×10^{-50} ***
Band - Bcar	1.94	0.0264	0.211
Bbav - Bcar	-12.14	3.2×10^{-34}	9.28×10^{-33} ***
Bbis - Bcar	0.9	0.185	0.738
Bbss - Bcar	-16.07	2.11×10^{-58}	8.87×10^{-57} ***
Bafz - Bgar	-11.03	1.43×10^{-28}	4×10^{-27}
Bam - Bgar	-13.3	1.18×10^{-40}	3.88×10^{-39} ***
Band - Bgar	3.7	0.00011	0.00176 **
Bbav - Bgar	-10.38	1.48×10^{-25}	3.71×10^{-24} ***
Bbis - Bgar	2.66	0.00395	0.0434 *
Bbss - Bgar	-14.31	9.39×10^{-47}	3.48×10^{-45} ***
Bcar - Bgar	1.76	0.0394	0.276
Bafz - Bkurt	-0.3	0.38	0.38
Bam - Bkurt	-2.58	0.00498	0.0498 *
Band - Bkurt	14.42	2.04×10^{-47}	7.74×10^{-46} ***
Bbav - Bkurt	0.34	0.368	0.735
Bbis - Bkurt	13.38	4.08×10^{-41}	1.39×10^{-39} ***

Bbss - Bkurt	-3.59	0.000166	0.00232**
Bcar - Bkurt	12.48	4.83×10^{-36}	1.45×10^{-34} ***
Bgar - Bkurt	10.72	4.04×10^{-27}	1.09×10^{-25} ***
Bafz - Bval	-7.36	9.08×10^{-14}	2.09×10^{-12} ***
Bam - Bval	-9.63	2.86×10^{-22}	6.87×10^{-21} ***
Band - Bval	7.36	9.22×10^{-14}	2.03×10^{-12} ***
Bbav - Bval	-6.72	9.18×10^{-12}	1.84×10^{-10} ***
Bbis - Bval	6.32	1.3×10^{-10}	2.47×10^{-9} ***
Bbss - Bval	-10.65	9.08×10^{-27}	2.36×10^{-25} ***
Bcar - Bval	5.42	2.94×10^{-8}	5.29×10^{-7} ***
Bgar - Bval	3.66	0.000124	0.00186 **
Bkurt - Bval	-7.06	8.52×10^{-13}	1.79×10^{-11} ***

Supplementary Table S3. Results of the ART ANOVAs, testing the effect of age category and sex on complement-mediated killing. Significance levels: * $p \leq 0.05$, ** $p \leq 0.01$, *** $p \leq 0.001$.

(3A) The high-sensitivity *Borrelia* group.

Effect	df	F-value	*p*-value
Age Category	5	1.4656	0.19963670
Sex	1	19.1766	1.4715×10^{-5} ***
Age Category × Sex	5	4.2831	0.00080765 ***

(3B) The medium-sensitivity *Borrelia* group.

Effect	df	F-value	*p*-value
Age Category	5	4.1158	0.0018468 **
Sex	1	2.5130	0.1158315
Age Category × Sex	5	1.7262	0.1346905

(3C) The low-sensitivity *Borrelia* group.

Effect	df	F-value	*p*-value
Age Category	5	6.31031	1.0202×10^{-5} ***
Sex	1	22.51654	2.6188×10^{-6} ***
Age Category × Sex	5	0.68695	0.63347

Supplementary Table S4. Wilcoxon rank sum test results comparing complement-mediated killing between sexes within each age category for high-, medium-, and low-sensitivity *Borrelia* groups. P-values adjusted using the Holm-Bonferroni method. Significance levels: * $p \leq 0.05$, ** $p \leq 0.01$, *** $p \leq 0.001$.

Sensitivity	Age Category	Raw p-value	Adjusted p-value	Higher Group
High	1	0.214	0.643	M
High	2	0.00254	0.0127 *	M
High	3	1.57×10^{-6}	9.43×10^{-6} ***	M
High	4	0.962	0.962	F
High	5	0.143	0.573	M
High	6	0.376	0.751	M
Medium	1	0.364	1	M
Medium	2	0.0233	0.14	M
Medium	3	0.307	1	M
Medium	4	0.52	1	M
Medium	5	0.595	1	M
Medium	6	0.111	0.555	F
Low	1	0.00831	0.0499 *	M
Low	2	0.0344	0.103	M
Low	3	0.0143	0.0716	M
Low	4	0.493	0.493	F
Low	5	0.075	0.15	M
Low	6	0.0255	0.102	M
